# Automated Cephalometric Points Marking System

**DOI:** 10.3390/diagnostics16111638

**Published:** 2026-05-27

**Authors:** Kaja Szwarczyńska, Eryk Kosmala, Maciej Antczak, Ivo Domagała, Barbara Biedziak, Jędrzej Musiał

**Affiliations:** 1 Poznan University of Medical Sciences, Department of Orthodontics and Craniofacial Anomalies, Fredry 10, 60-812 Poznan, Poland; 2 Institute of Computing Science, Poznan University of Technology, Piotrowo 2, 60-965 Poznan, Poland

**Keywords:** medical decisions, algorithms, AI, decision support system, cephalometric analysis, deep learning, image processing

## Abstract

**Background/Objectives:** Modern artificial intelligence methods are increasingly used in medical and dental image analysis to support diagnosis and treatment planning. In orthodontics, automatic detection of cephalometric landmarks from X-ray images remains a challenging and clinically relevant task. **Methods:** This study proposes a multi-model approach for cephalometric landmark detection based on the ALD algorithm and three derived models trained with extended image augmentation techniques. The applied augmentations, including contrast and negative transformations, improved the detection of specific anatomical landmarks. The final detection strategy integrates outputs from all four models, selecting the most accurate prediction for each landmark based on historical performance results. **Results:** The proposed method was evaluated on real datasets. It achieved a mean radial error (MRE) of 2.12 mm compared to 2.26 mm for the baseline model, and a successful detection rate (SDR) of 72.22% within a 2.5 mm threshold compared to 68.87% for the baseline model. **Conclusions:** The results demonstrate that the ensemble-based approach improves landmark detection accuracy and has the potential to support clinical orthodontic workflows.

## 1. Introduction

### 1.1. Preliminary Information

Medical decisions are increasingly based on modern computer solutions in medicine and dentistry. Artificial intelligence applies to X-rays, computerized tomography (CT), and magnetic resonance imaging (MRI) image analysis. Artificial intelligence is becoming indispensable in daily clinical practice, supporting physicians in diagnosis and treatment planning. By analyzing clinical data, including images of anatomical structures, AI systems assist in diagnosing diseases and establishing treatment plans. In dentistry, artificial neural networks help analyze radiographic images of the dentition to detect carious cavities or periodontal disease. Researchers Tuzolf et al. used a spliced neural network to analyze pantomographic images to detect and number teeth automatically. The algorithm’s sensitivity was comparable to the diagnosis made by doctors [1]. Researchers from South Korea described the use of neural networks with high efficiency in detecting dental abnormalities and diagnosed an extra tooth in the jaw [2]. Meanwhile, Zaborowicz et al. used artificial neural networks to assess a patient’s metric age by analyzing a pantomographic photo [3]. Cieslinska and co-authors proved the effectiveness of diagnosing the position of the second premolar using artificial neural networks [4].

### 1.2. Motivation

Cephalometric image analysis is indispensable to malocclusion diagnosis and orthodontic treatment planning. Malocclusion is associated with abnormalities in the morphology of the bony structures of the facial part of the skull and the position of the teeth. The precursors of cephalometric analysis were Broadbend, Hofrath, Rehak, and Korkhaus, who defined various measuring points, planes, and angles. With advances in technology, cephalometric analysis evolved. More than two hundred different measurement methods have been developed. Each is based on its definitions of points and reference lines. The orthodontic cephalometric analysis will determine the maxilla’s position, the mandible’s position, facial proportions, vertical relationships, positions of molars and incisal teeth (maxilla and mandible), and correlation of bony, dental, and soft tissue structures. Recently, advances in cephalometry have been induced by the development of imaging systems and computational computer analysis. Using machine learning to diagnose reference points significantly saves the physician’s time required for image analysis. The availability of multiple computational cephalometric analyses makes it possible to select the type of measurements and create personalized diagnostics from them.

While the computer systems introduced for cephalometric analysis calculate individual angles, measure segment lengths, and determine ratios, defining measurement points in most analyses relies on the doctor’s determination of points. However, most existing approaches rely on a single landmark detection model, which assumes uniform performance across all anatomical landmarks. This paper presents image analysis and artificial intelligence algorithms to determine reference points in cephalometric analysis. In practice, this assumption is often not satisfied, as individual landmarks differ in terms of visibility, contrast, and anatomical variability, which leads to inconsistent detection accuracy across points.

Machine learning-based models confirmed their usefulness in solving very diverse practical problems, e.g., smartphone aspect-based rating prediction [5], chess piece recognition [6], and traffic forecasting in dynamic optical networks [7]. Moreover, deep neural networks can successfully compete with experts in biologically inspired applications [8,9,10]. However, the success of sophisticated image processing models generally depends on reliable feature extraction and classification [11,12]. A single model/classifier often fails to ensure the expected generalization ability. Thus, an ensemble of models/classifiers is proposed, where every model/classifier focuses on different feature sets selected independently [13]. Their results are combined with other commonly used techniques, e.g., a random forest, to ensure higher accuracy than any independently considered model [14]. In contrast to conventional ensemble approaches, the method proposed in this study performs landmark-wise model selection, allowing different models to be assigned to specific anatomical landmarks.

### 1.3. Research Objectives

The aim of this study was to investigate the impact of image augmentation on the accuracy of cephalometric landmark detection. In particular, the study evaluates how augmentation-driven diversity can be exploited through a multi-model framework to improve detection performance. The proposed approach involves the development of four distinct models, each trained on a different image dataset generated using specific augmentation techniques (as described in Section 3.1). Unlike conventional approaches based on a single model, the proposed method distributes the prediction task across multiple specialized models and selects the most accurate prediction for each landmark. This strategy enables the construction of a system with enhanced detection accuracy by accounting for landmark-specific variability.

### 1.4. Description of Work

The rest of the work is presented as follows. In Section 2, an introduction to the topic from the literature (state-of-the-art) was made. Section 3 describes the materials and methods, including the datasets used, the base model, and the proposed training and testing methodology. Section 4 presents the results of the conducted experiments. Section 5 discusses the obtained results in detail, highlighting their implications and limitations. Section 6 provides a concise conclusion summarizing the main observations of the study. Finally, Section 7 contains information regarding the availability of the source code and implementation details.

## 2. State-of-the-Art

Nowadays, several approaches are available for the localization of cephalometric landmarks with sufficient accuracy in lateral cephalograms. The most commonly used techniques are deep learning end-to-end networks. Generally, CNNs are used to learn features of the anatomical context. In addition, these networks are usually equipped with attention mechanisms to strengthen performance. Recent developments in medical image analysis have emphasized the increasing relevance of hybrid architectures that integrate convolutional neural networks with transformer-based components. For instance, a recent study [15] introduced a deep learning framework enhanced with a transformer module for medical image denoising, showing that the combination of local feature extraction and global contextual modeling leads to improved image quality and better preservation of structural details. Although the study was conducted on mammographic data, the reported results suggest that integrating convolutional and attention-based mechanisms can improve the identification of subtle anatomical features. Consequently, such approaches are also applicable to cephalometric landmark detection, where both fine-grained local information and broader anatomical context play a crucial role.

The work in this section includes a broad review of the most current methods and scientific research that can be applied to solve the posed problem of automated cephalometric point determination [16,17,18,19,20,21,22]. Particularly noteworthy are methods that can form the basis for further work, leading to the development of dedicated solutions to the problem posed in the title. This section summarizes the existing knowledge and indicates the applied approach to the issue under consideration. At the same time, we are constantly mindful of the great importance of the research’s applicability. This places significantly extended demands on the problem in terms of the calculation time and accuracy of the indicated solutions. Not only is the distance from the correctly marked point essential, but also the type of errors. All of these aspects should be realistically evaluated before being applied in clinical practice, which is also addressed in the discussion of the computational experiment results.

Chen et al. [16] proposed a comprehensive deep learning system. The task was to detect cephalometric points as precisely as possible while maintaining automation and efficiency. The network architecture includes three modules arranged sequentially: a feature extraction module, an attentive feature pyramid fusion module (AFPF), and a prediction module. The feature extraction module uses a 19-layer convolutional neural network, VGG-19 [23,24], as the backbone. The authors emphasized that features extracted by different layers of the neural network have varying resolutions and semantic meanings. Identification of landmarks at boundaries requires high-resolution and detailed structural information, while the identification of landmarks in central regions requires deeper semantic information. Different features were combined to achieve a high-resolution and semantically enhanced fusion function. Each landmark was associated with a relevance value linked to specific features, and a self-attention mechanism was used to learn the weights assigned to these features. The prediction module uses a combination of heat maps and offset maps to perform pixel-level regression more efficiently. The precision of detecting landmarks in Dataset 1 [25] was 86.67% within a clinically acceptable range of 2.0 mm, with an average error of 1.17 mm. For Test Set 2 [25], 75.05% precision was obtained within the same range, with an average error of 1.48 mm. The method outperformed classical solutions by 7–11%.

Qian et al. [17] proposed a novel multi-headed neural network, CephaNN, for detecting cephalometric landmarks. CephaNN is an end-to-end heatmap-based network consisting of multi-head and attention components for coarse-grained detection. In the multi-head part, two U-Net-shaped subnetworks are used to learn features from different perspectives, and indirect supervision is applied to speed up convergence. Based on this module, the attention component generates feature maps with multiple attention mechanisms to improve detection results. A region-enhancing (RE) loss function was introduced to improve performance in critical regions. Studies on Dataset 1 and Dataset 2 showed that CephaNN achieved detection accuracies of 87.61% and 76.32%, respectively, within a clinically acceptable range of 2.0 mm, with average distance errors of 1.15 mm and 1.43 mm. Additional experiments on extended anatomical classification and a 75-point real-world dataset confirmed the effectiveness and robustness of CephaNN.

Oh et al. [18] hypothesized that deep learning enables more precise generalization when local characteristics and anatomical context are analyzed simultaneously during training. Their work presented a framework called deep anatomical context feature learning (DACFL), which enables the simultaneous learning of local features and anatomical context. The method includes two main components: a local feature perturbator (LFP) and an anatomical context (AC) loss function. The LFP modifies local features of cephalometric images based on anatomical distribution, enhancing global feature representation. The AC loss function incorporates geometric relationships between landmarks, allowing the model to better capture spatial dependencies. This is particularly important in noisy images where local pixel information may be insufficient. The DACFL approach enables the network to learn both contextual and local representations. Experimental results showed that DACFL significantly improved the error detection rate (EDR) in the range of 2.5–4 mm. The method achieved detection accuracies of 86.20% and 75.89% within 2.0 mm, with average errors of 1.18 mm and 1.45 mm, respectively.

Quan et al. [19] pointed out that annotating large medical imaging datasets requires significant time and expertise from radiologists. To address this limitation, they proposed a few-shot learning approach capable of achieving competitive results with only a small number of labeled samples. A key component is the sample/photo selection (SCP) method, which identifies the most informative images for annotation. SCP consists of three stages: self-supervised training for feature extraction, key point proposal to identify regions of interest, and estimation of representativeness scores to select optimal samples. Experimental results showed that SCP reduced the mean radial error by 14.2% (from 3.595 mm to 3.083 mm) for cephalometric datasets and by 35.5% (from 4.114 mm to 2.653 mm) for hand X-ray datasets. The method performs particularly well when only a small number of images (10 or fewer) are annotated, but its effectiveness decreases when larger annotated datasets (25 or more images) are available.

Fully automatic landmark annotation (FALA) [20] refers to a system for the rapid and accurate placement of anatomical landmarks. Originally developed for orthopedic applications, it was later adapted for cephalometric analysis. The system uses random forests (RF) to detect the position, scale, and orientation of the skull, followed by a constrained local model (RFRV-C-CL) for precise landmark localization. The first stage ensures robustness to variations in image acquisition, while the second enables the accurate placement of all 19 landmarks. The average processing time was 24 s, reduced to 3 s in the improved version.

The FALA system achieved a point-to-point error (PE) of 1.7 ± 0.02 mm for 95% of 400 images. In comparison, manual interobserver variability yielded a PE of 2.2 ± 0.03 mm. Intraobserver variability was 1.7 ± 0.01 mm for physician 1 (senior) and 0.9 ± 0.01 mm for physician 2 (junior). Additional experiments using physician 2’s annotations as ground truth showed that higher-quality annotations significantly improved the model performance, highlighting the importance of training data quality.

FALA achieved detection rates of 84.7% and 96.3% within 2.0 mm and 4.0 mm thresholds, respectively, outperforming manual interobserver analysis (62.1% and 85.0%). However, performance varied depending on the landmark. For example, the L10 landmark (Gonion) showed lower accuracy, with a point-to-point error (PEL) of 2.69 ± 0.12 mm and detection rates of 50.25%, 57.00%, 65.25%, and 79.75% for thresholds of 2.0 mm, 2.5 mm, 3.0 mm, and 4.0 mm, respectively. The largest manual discrepancies were observed for L16, with a PEL of 6.57 ± 0.18 mm. In contrast, FALA achieved better results for L16 (soft tissue pogonion), with a PEL of 1.23 ± 0.06 mm for the model trained on physician 2’s annotations and 3.87 ± 0.20 mm for the model trained on physician 1’s annotations.

In addition to methodological advancements, recent studies have also investigated the clinical applicability of artificial intelligence in cephalometric analysis. A comparative study [26] evaluated the performance of fully automated AI-based systems against manual and semi-automatic approaches. The findings showed that while AI-driven methods substantially reduce the time required for analysis, their accuracy may vary across different anatomical landmarks, with certain points remaining difficult to detect reliably. Importantly, the study demonstrated that combining AI-generated predictions with expert refinement yielded results comparable to those obtained using traditional methods. These observations indicate that despite significant progress, fully automated solutions still require further improvements in terms of accuracy and robustness, thereby justifying continued research in this field.

## 3. Materials and Methods

### 3.1. Dataset Description

The images used in the experiments came from two different collections. The first set of images used was provided as part of the article “Fully Automatic System for Accurate Localization and Analysis of Cephalometric Landmarks in Lateral Cephalograms” [20] and consists of a collection of 400 images in BMP format. The size of each photo is 1935 × 2400 pixels. Each of the photos has information on the marking of 19 cephalometric points, taken by a novice and an experienced dentist. For our experiments, we used the markings made by the more experienced expert. We divided the collection of images into a training set, which contained 300 images, and a test set, in which we included 100 images—corresponding to a 75:25 train–test split. The training set itself was divided 50:50 into the actual training set and the validation set used in the learning process.

The second collection contained 1430 images in JPG format. The resolution of each image is 1005 × 1271 pixels. We have information on about 83 cephalometric points the dentists marked for each image. In this experiment, we divided the training and test collection at a ratio of 85:15. The variation in train–test split ratios is a consequence of the differing dataset sizes. For the larger dataset, a higher proportion of samples was assigned to training and validation, which was feasible and appropriate given its greater volume. The training collection consisted of 1000 training images and 215 validation images. The remaining 215 images were used as a test dataset.

**Figure 1:**
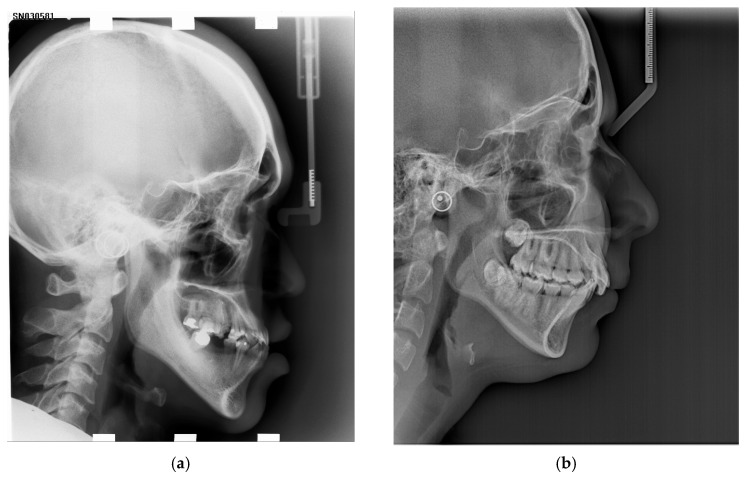
Images from the datasets described in this study: (**a**) photo from the first collection (BMP format, size 1935 × 2400 pixels); (**b**) photo from the second collection (JPG format, size 1005 × 1271 pixels).

The photo in Figure 1a is from the first collection of X-rays. The photo in Figure 1b, on the other hand, shows an image that is part of the second collection of images. The photos included in each collection differed in contrast levels and brightness, as observed in the figures below.

### 3.2. Data Augmentation

For each of the two datasets described in Section 3.1, three additional image subsets were generated per dataset, resulting in a total of four subsets for each dataset, each differing in the applied augmentation strategy. The first subset consisted of the original, unmodified images and served as a baseline for comparison. The remaining subsets were created using image augmentation techniques implemented with the Python (3.10) Pillow (9.2.0) library. The second subset comprised negative images obtained through pixel-wise intensity inversion, where each pixel value was transformed to its complementary intensity. This operation effectively enhanced structures that may be less visible in standard radiographs, which was motivated by expert observations.

The third subset was generated by modifying the contrast of the original images. This process involved adjusting the global contrast level to emphasize anatomical structures, thereby potentially improving the visibility of certain cephalometric landmarks. The fourth subset combined both transformations. Specifically, each image was first converted into its negative form, and subsequently, contrast enhancement was applied. This combined approach aimed to exploit the advantages of both intensity inversion and contrast adjustment. As a result, four distinct subsets were obtained for each of the two primary datasets. Each subset (i.e., original, negative, contrast-enhanced, and combined) was treated as an independent dataset and used to train a separate model; no mixing of different transformation types occurred within a single model. The train–validation–test split was then applied independently within each subset (not across combined augmented data), following an identical deterministic indexing scheme for all subsets derived from a given original dataset. Consequently, corresponding images across different subsets (e.g., an original image and its transformed versions) were consistently assigned to the same partition (training, validation, or test) within their respective datasets. This ensured that no transformed version of a given image appeared in a different data subset than its counterparts, thereby preventing data leakage between the training, validation, and test sets.

Consequently, for each dataset variant, all corresponding models shared the same dataset size and identical partitioning scheme as their respective original dataset. The only difference between the subsets lies in the applied augmentation strategy. Therefore, each model was trained on data of the same cardinality and split structure, ensuring that performance differences were attributable solely to the augmentation type rather than variations in dataset size or sampling. These subsets were then used independently in the training, validation, and testing processes of the developed models.

### 3.3. Base Model

The proposed backbone follows Chen et al. [16]. The architecture consisted of three main components: a feature extraction module, an attentive feature pyramid fusion (AFPF) module, and a prediction module. In the feature extraction stage, VGG-19 was employed as the backbone network to generate hierarchical feature representations.

The feature maps produced by the first module are forwarded to the AFPF module, where they are processed to produce a tensor of size (3n, h, w), where 3n corresponds to n heat maps and 2n offset maps, while h and w denote the spatial dimensions of the input image. Heat maps are used to localize approximate regions of landmark positions, whereas offset maps act as regressors that refine the exact coordinates of the cephalometric points. The authors observed that different landmarks attend to different feature representations; therefore, a self-attention mechanism was introduced to learn adaptive weights for each landmark, defined as:

(1) $$a_{k}=softmax(W_{k1}tanh(W_{k2}\tilde{F})\;),$$

where $a_{k}$ is an attention matrix composed of three attention vectors (one for the heat map and two for the offset maps). $W_{k1}$ and $W_{k2}$ are trainable matrices presented by fully connected layers without bias. $\tilde{F}$ is obtained by operations of average pooling and reshaping that transfers F. For each landmark, the attention weights $a_{k}$ are applied to the feature pyramid F through channel-wise multiplication, resulting in weighted feature representations:

(2) $$F_{k}=c(a_{k}⨂\;F),$$

where $F_{k}$ consists of three weighted feature maps, each preserving the same spatial dimensions as the original feature pyramid F. Here, ⨂ denotes channel-wise multiplication, and c represents the number of channels. In the prediction stage, the final landmark locations are obtained by combining the heat maps and offset maps generated by the AFPF module. The loss function for heat maps, $L_{h}$, is defined as a mean logistic loss between the predicted heat maps and the ground truth. The loss function $L_{o}$ is defined to be the L1 loss between the predicted offsets and the target. The overall loss function is formulated as a weighted sum:

(3) $$L\left(\theta \right)=\alpha L_{h}\left(\theta \right)+(1-\alpha )L_{o}(\theta ),$$

where α is a balancing coefficient empirically set to 2/3. Importantly, the offset loss is computed only for pixels within a predefined radius R, rather than over the entire image domain, which focuses the learning process on relevant regions surrounding each landmark [16].

### 3.4. Models and Algorithms Design

During the experiments, we trained four models for each dataset. As discussed in Section 3.3, the foundation of each model was based on the solution proposed by the authors of the ALD (Anatomic Landmark Detection) tool. The first set of four models was used to indicate 19 cephalometric points on the X-ray. Due to a different training set, the second set of the models indicated 83 cephalometric points. The first model for both collections was trained on a set of unmodified photos, which were loaded and scaled to a size of 800 × 640 pixels. For training the next (second) model, negatives created for both datasets were used and scaled to the same size. This decision was made based on input from a collaborating expert, who found specific points easier to observe on the negative images. This observation motivated the inclusion of negative-image augmentation as a complementary training strategy, and its impact on landmark detection performance was empirically evaluated. These images were loaded using the OpenCV (4.7.0.72) library. The image negatives were obtained by a simple operation of 255 − image, where image was a variable that contained the previously loaded individual image.

The third model was trained for each collection of images with modified contrast and brightness levels. For this purpose, the α parameter responsible for the contrast level was set to 0.75, while the β parameter, which controls the image’s brightness, remained at 0. The values of these parameters were determined through a series of experiments. The rationale behind these experiments stemmed from the hypothesis that controlled modifications of image contrast and brightness may enhance the perceptibility of subtle anatomical structures in regions where landmark localization is inherently difficult. By adjusting the intensity distribution, it was expected that edge definitions and local gradients would become more distinguishable, potentially improving the robustness of feature extraction and landmark detection. The experimental results presented in Table 1 indicate that such preprocessing can positively influence the performance of the models for selected landmarks, which supported the decision to include contrast-based augmentation as part of the training pipeline.

The last model was trained on a set of negative images whose contrast level was then altered. During the preparation of this dataset, the parameters and methods used for the two previously described models were applied. The images were first subjected to a negative transformation, followed by contrast adjustment. The contrast scaling parameter α was set to 0.75, while the brightness offset β remained at 0.

### 3.5. Multi-Model Selection Engine

Each model was trained on dataset variants described in the previous sections. The detailed baseline architecture and data augmentation strategies were introduced in Section 3.2, Section 3.3 and Section 3.4. During inference, all trained models are executed in parallel, and their outputs are combined within the proposed selection engine.

The selection mechanism determines the final coordinates of each landmark based on statistical performance obtained during validation, assigning the most reliable prediction for each point from among the available model outputs. The Euclidean distance metric is used as the primary criterion for evaluating prediction accuracy during this selection process. The key differences between the trained models are summarized in Table 2. Separate model sets were trained for each of the two X-ray datasets, as described previously.

**Figure 2:**
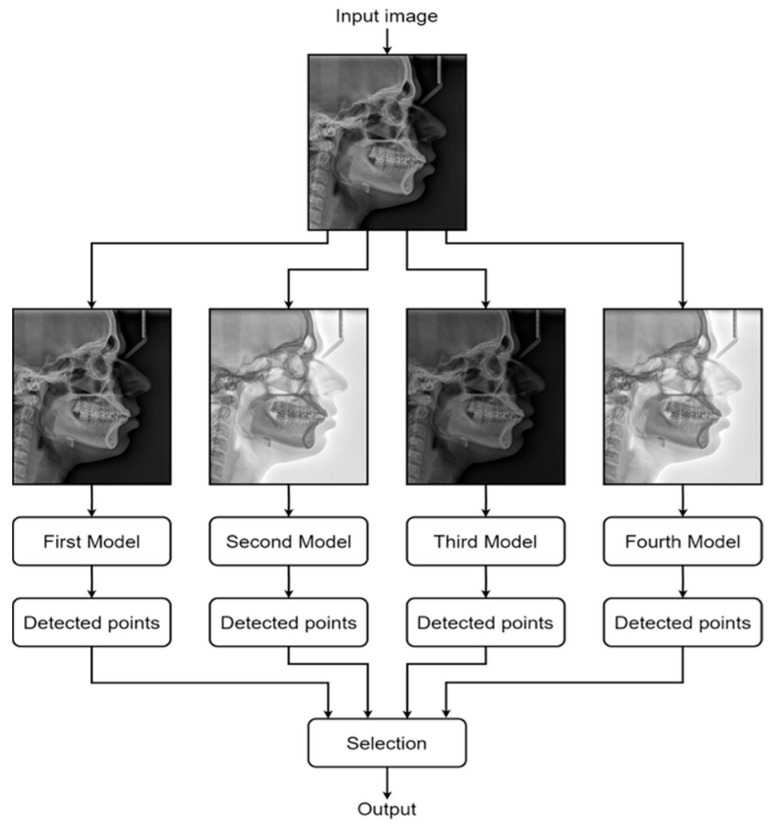
Diagram illustrating the operation of the proposed engine—the original input image is forwarded to one of four models: ALD; CNeg (contrast-negative augmented); Con (contrast augmented); and Neg (negative augmented). The final output is obtained as a combination of predictions from the individual models, where each landmark is selected from the model that has demonstrated the highest historical accuracy for that specific point.

The previously mentioned detection engine was created using these prepared models. Figure 2 presents the overall workflow of the proposed engine. As described above, the engine takes an image as input, duplicating each copy undergoing specific augmentations. The respective images are fed into individual models, each making separate predictions.

Each model predicts all cephalometric landmarks independently. However, the final position of a given landmark is selected from the model that has historically demonstrated the highest accuracy for that specific landmark. This selection is based on statistical performance evaluated during validation experiments, where each model is assessed individually for its localization error. Consequently, for each landmark, the prediction is taken from the model that achieved the lowest error metrics for that particular point, ensuring that the final output leverages the most reliable model on a per-landmark basis. An overview of the workflow of the proposed approach is provided in Table 3.

### 3.6. Model Evaluation Metrics

The primary metrics used for evaluating and comparing the performance of the individual models were the mean radial error (MRE) and success detection rate (SDR). These metrics are widely adopted in the assessment of cephalometric landmark detection systems because they quantify both the average localization accuracy and the proportion of predictions that fall within clinically acceptable error thresholds.

Mean radial error (MRE) measures the average Euclidean distance between the predicted landmark coordinates and the corresponding ground truth annotations. For a set of N landmarks, the radial error for the i-th landmark is defined as the Euclidean norm between the predicted position ${\hat{p}}_{i}=({\hat{x}}_{i},{\hat{y}}_{i})$ and the ground truth position $p_{i}=(x_{i},y_{i})$. The MRE is then computed as:

(4) $$MRE=\frac{1}{N}\sum_{i=1}^{N}d_{i}\;\;\;\;\;\;\;\;$$

(5) $$d_{i}=\sqrt{{\left({\hat{x}}_{i}-x_{i}\right)}^{2}+{\left({\hat{y}}_{i}-y_{i}\right)}^{2}},$$

where $d_{i}$ is the radial error for the i-th landmark, and N is the total number of landmarks. A lower MRE value indicates a higher overall localization accuracy.

The success detection rate (SDR) quantifies the percentage of landmarks for which the radial error is within a specified threshold (e.g., 2.0 mm, 2.5 mm, 3.0 mm). It is defined as:

(6) $${SDR}_{r}=\frac{\#\{i\;:{\;d}_{i}\;\leq \;r\}}{N},$$

where $\#\{i\;:d_{i}\leq r\}$ denotes the number of landmarks whose radial error does not exceed the threshold r. SDR is typically reported at multiple thresholds to reflect clinical tolerance levels, with higher values indicating a greater proportion of accurately localized landmarks.

Together, MRE and SDR provide a comprehensive evaluation of model performance: MRE assesses the average prediction accuracy, while SDR captures the reliability of landmark detection within clinically relevant tolerances.

### 3.7. Training and Validation

For the first collection of X-rays, a learning process was carried out for each model on the same set of 300 images. The only differences were in the image augmentation, following the description provided in Section 3.2. The learning process took 350 epochs for each model. MRE and SDR values were recorded for each epoch (considering different radius values). The learning process for the second set of images involved training each model on the same set of 1215 images. As for the first collection, the only variations occurred in image augmentation. Each model was subjected to a learning process that lasted 350 epochs. MRE and SDR values were recorded for each epoch, considering different radius values. All parameters used during the training process of the individual models are summarized in Table 4.

**Figure 3:**
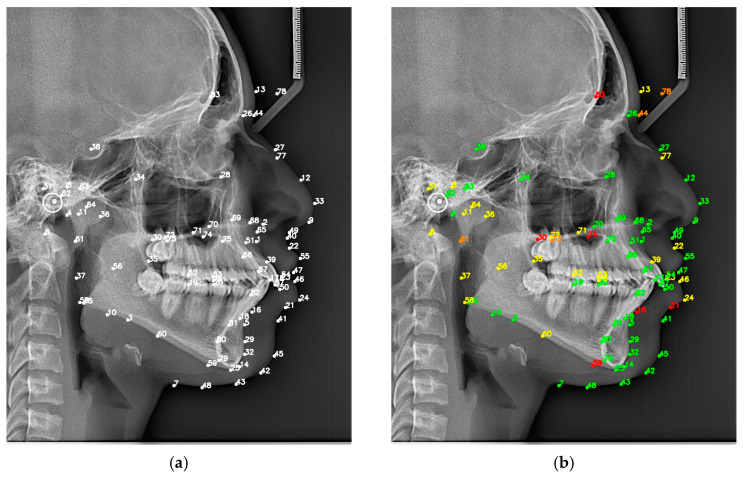
X-ray images with annotated cephalometric landmarks: (**a**) X-ray photos with manually marked cephalometric points; (**b**) X-ray photos with cephalometric points marked by algorithm.

MRE and SDR values were recorded for each point of each image. Based on these data, the models were subsequently evaluated according to their ability to predict individual points. In Figure 3a, a photo with the actual distribution of cephalometric points is presented. Meanwhile, in Figure 3b, the points have been marked according to the results of the engine’s predictions.

The color of the point indicates the detection accuracy. The meanings of individual colors are as follows:

- Green—error relative to the original position less than 2.5 mm;
- Yellow—error relative to the original position less than 5 mm;
- Orange—error relative to the original position less than 7.5 mm;
- Red—error relative to the original position less than 10 mm.

### 3.8. Test Environment

The training and subsequent testing of the models were conducted on a shared machine specifically designed for machine learning tasks. The machine was equipped with an NVIDIA RTX™A6000 graphics card (NVIDIA Corporation, Santa Clara, CA, USA) containing 48 GB of dedicated GDDR6 GPU memory. Furthermore, it featured an Intel^®^Xeon^®^E5-2609 v4 processor (Intel Corporation, Santa Clara, CA, USA) running at 1.70 GHz, with 20 MB of Intel^®^ Smart Cache.

The machine was equipped with 64 GB of RAM. The operating system used on the machine was Ubuntu version 20.04. All calculations and tests were performed in Python 3.10 using the PyTorch 1.13.0 (cu117) library. Utilizing the NVIDIA-provided CUDA 11.7 toolkit facilitated faster computations, including tensor operations.

## 4. Results

The previous research and experiments have mainly focused on specific precision ranges. These distances, which indicate the deviation of the point determined by the algorithm from the mark plotted by the dentist’s expert, typically range from 2.0 mm to 4.0 mm. Based on the input of co-authors with extensive medical experience who work daily in describing and analyzing cephalometric images, it has been suggested that the most suitable representation would be using a precision range of 2.5 mm. Marking a point within this range provides an excellent approximation for accurate image analysis, and any required manual corrections can be completed quickly and efficiently. Table 5 includes the aggregated test results.

### 4.1. Computational Experiment—Alpha

The first computational experiment was conducted for the first dataset, described earlier in Section 3.1. The study involved determining 19 cephalometric points for 100 test images. The study used 4 algorithms/models: ALD, Con—contrast augmented, Neg—negative augmented, and CNeg—contrasted-negative augmented, which are described in more detail in Section 3.4. Therefore, it can be noted that the testing process alone (excluding the learning process) determined 7600 points. This experiment aimed to demonstrate new algorithmic approaches for a data model known in the literature. The summary information of the conducted experiment is presented in Table 5.

It should be noted that none of the solutions was dominant. Each of them achieved the best SDR score (the highest percentage of accurately described, for a given point, images from a set of 100 for an assumed precision rate). MRE was closely correlated with SDR, indicating the excellent stability of all algorithms. There is no situation in which an algorithm obtaining high SDR in certain situations suddenly makes fatal determinations (which would affect the significant increase in MRE). All algorithms obtained very low SDRs for some points (e.g., 3 and 6). This may have to do with the very characteristic nature of these points. Looking at the global picture, the best approach is to identify the dominant algorithm (or algorithms) for each successive point, and point to its marking as the answer for the analyzed image.

### 4.2. Computational Experiment—Beta

The second computational experiment was conducted for the second dataset, described earlier in Section 3.1. The study involved determining 83 cephalometric points for 215 test images. The study used the same 4 algorithms/models as before: ALD, Con—contrast augmented, Neg—negative augmented, and CNeg—contrasted-negative augmented. Therefore, it can be noted that the testing process alone (excluding the learning process) determined 71,380 points. This was an almost 100 times higher number compared to the smaller dataset. Table 6 contains the detailed results (aggregated) of this computational experiment.

Analyzing the results of this pervasive computational experiment, it was necessary to confirm the high validity of using our proposed solution. It can be perceived as a pseudo-hyper-heuristic, indicating for each of the analyzed points (out of their complete set of 83) which of the 4 integrated solutions should be used to obtain the best final results. Applying each of the algorithms separately, we obtained the SDR (the SDR of a single point met, if and only if, all 215 test measurements fell within the indicated precision range—so this is a very restrictive assumption) for all 83 points at levels in turn: 68.87%, 70.22%, 47.65%, and 68.62%, respectively, for the baseline model, the negative-image model, the contrast-adjusted model, and the combined negative-and-contrast model. Using the indicated combined approach, our best CNeg algorithm’s result improved to 72.22%. The need for the concurrent analysis of MRE should be strongly emphasized, which, among other things, makes a critical observation. A score of 72.22% does not mean that almost 28% of the points (23 points) were mislabeled and are worthless. The result indicates that 60 points are even subject to automatic acceptance, and the subsequent points are (as further analysis indicates) very close to the designated precision point. They, therefore, require conditional approval by the expert analyzing the result or a slight improvement in position.

It is therefore necessary (besides the SDR analysis) to analyze the MRE and the SDR, since this is the average error for a given point after all 215 measurements. Thus, for even one of the images, the determination exceeds the precision range, and the SDR will be zero (for that point), but the MRE for all samples will be much lower than the assumed precision threshold. For the standard indicated precision threshold (2.5 mm), 73.49% of the points (61) were within this range—referring to the regency ALD algorithm, we obtained a value of 67.47% (56 pts) for it. If we increased the acceptable marking range by only 0.5 mm (i.e., to 3.0 mm), we found that as many as 93.98% (78) of the points were (in terms of MRE) within the indicated range. Increasing the precision range further by another 0.5 mm (to 3.5 mm), we obtained a result of 97.59% (81 pts). Complete coverage for all 83 points was obtained with the precision range extended to 4.62 mm.

Our solution/system (3 of which are our new proprietary solution) achieved higher accuracy than the existing models provided in the literature. The system combined 4 different algorithms achieved (comprehensive experiment with the 83 cephalometric points) an average point-to-point error (MRE) of 2.12 mm (compared to the 2.26 mm for the ALD), and 72.22% of landmarks were located (SDR) within the clinically accepted precision range of 2.5 mm (compared to the 68.87% for the ALD).

## 5. Discussion

Classic optimization and scheduling problems that can be found in the market are, in a very general way of thinking, based on a better, more efficient plan for performing the work to achieve the desired result [27,28,29,30]. For these types of problems, the input is known; we know (at some level of generality) what actions we can and want to take, and the main question is how to take these actions (in what order, proportion, etc.) to obtain the best result (objective function). The problem addressed in this paper is different, although it can just as boldly be categorized top-down as optimization. The input data (X-ray images) are known. Many efficient results are known (described samples—X-ray images with marked cephalometric points). The question remains: how do we teach the algorithm to automatically perform the assigned tasks (marking points) to go from the data level to the result (an acceptable level for a dentist)?

Here, we first prepared a diverse collection of images. Next, commonly used approaches were selected and applied for specialized learning. Based on them, we trained, validated, and precisely evaluated the models in an iterative manner. Partial results showed us that wider experimenting with learning parameter values is required. We confirmed that our models achieved higher accuracy than the existing models in the literature. The service achieved (comprehensive experiment with the 83 cephalometric points) an average point-to-point error (MRE) of 2.12 mm (compared to the 2.26 mm for the ALD), and 72.22% of landmarks were located (SDR) within the clinically accepted precision range of 2.5 mm (compared to the 68.87% for the ALD). In the future, we will perform a high-scale hyperparameter optimization.

### Limitations and Future Work

The proposed approach is subject to several limitations. First, the availability of publicly accessible and sufficiently large cephalometric datasets remains limited, which constrains further model training and may affect generalization performance. Additionally, the detection of certain landmarks may be challenging in patients presenting significant craniofacial abnormalities, where anatomical structures are less clearly defined and exhibit higher variability. Finally, the proposed system is intended to serve as a decision-support tool rather than a fully autonomous solution, and therefore requires expert supervision to verify, and if necessary, refine the predicted landmark positions. Future work should focus on expanding the size and diversity of available datasets, as well as improving the robustness of the models to better handle anatomical variability and different imaging conditions.

## 6. Conclusions

The use of image augmentation to generate distinct datasets, and consequently to train multiple prediction models, enables the development of a composite system that employs individual sub-models for detecting specific subsets of cephalometric landmarks. This approach improves the overall accuracy of landmark prediction compared to a single model trained solely on the original, unmodified dataset.

The results also suggest that image augmentation has a meaningful impact on the detectability of certain cephalometric landmarks. Therefore, the appropriate application of augmentation techniques can enhance the performance and reliability of the proposed tool, making it more suitable for use by dental specialists.

## 7. Availability

The code used for training and testing the models discussed in this study is available in a GitHub repository. The corresponding link has been provided in the Supplementary Materials.

## Figures and Tables

**Table 1:** Effect of contrast and brightness levels on results returned by the model.

Contrast	Brightness	MRE [mm]	SDR 1 mm	SDR 2 mm	SDR 2.5 mm	SDR 3 mm	SDR 4 mm
0.5	0	1.6805	0.5749	0.7644	0.8211	0.8631	0.9131
0.5	25	3.1376	0.5789	0.7368	0.8013	0.8302	0.8644
0.5	50	2.7775	0.5986	0.7644	0.8026	0.8355	0.8723
0.75	0	1.6426	0.6776	0.8552	0.8986	0.9171	0.9342
0.75	25	1.5952	0.5526	0.7789	0.8434	0.8763	0.9157
0.75	50	1.4771	0.5881	0.7749	0.8381	0.8815	0.9211
1.5	0	3.1145	0.5842	0.7118	0.7394	0.7592	0.8000
1.5	25	3.4609	0.5657	0.7368	0.7855	0.8197	0.8631
1.5	50	5.0996	0.5723	0.7013	0.7407	0.7684	0.8105

SDR [%], successful detection rate within a given radius; MRE [mm], mean radial error.

**Table 2:** Summary of model design and key characteristics.

Model	Input Data Type	Augmentation	Parameters	Key Design Motivation	Expected Impact
Model 1 (ALD)	Original X-ray images	None	Image size: 800 × 640	Baseline model trained on unmodified data	Reference performance
Model 2 (Neg)	Negative images	Pixel inversion (255 − image)	Image size: 800 × 640	Expert knowledge: some landmarks more visible in negative images	Improved detection of selected landmarks
Model 3 (Con)	Contrast-adjusted images	Contrast and brightness modification	Image size: 800 × 640 α = 0.75, β = 0	Empirical optimization of image visibility	Enhanced feature representation
Model 4 (CNeg)	Negative + contrast-adjusted images	Combined augmentation (negative + contrast)	Image size: 800 × 640 α = 0.75, β = 0	Combination of beneficial transformations	Further improvement in landmark detection

The study tackled 4 algorithms: ALD; CNeg, contrasted-negative augmented; Con, contrast augmented; Neg, negative augmented.

**Table 3:** Overview of the proposed detection engine workflow.

Component	Description
Input	Single X-ray image
Preprocessing	Image duplication and augmentation (original, negative, contrast, combined)
Models	Four independently trained models (same architecture, different datasets)
Prediction	Each model predicts all landmarks
Selection strategy	Each landmark is selected from the model with the highest statistical accuracy

**Table 4:** Parameters used during the training of the individual models.

Parameter	Value
batchSize	1
landmarkNum	19/83 depending on the dataset used for training the model
image_scale	(800, 640)
epochs	350
R1, R2	41

**Table 5:** Experimental results involved the detection of 19 cephalometric points across 100 test images, reporting the percentage of correctly localized points within a 2.5 mm threshold and the mean deviation from the ground truth (in mm).

pt	SDR 2.5 mm [%]				MRE [mm]			
	ALD	CNeg	Con	Neg	ALD	CNeg	Con	Neg
1	99	99	99	99	0.69	0.74	0.65	0.59
2	92	94	92	92	1.11	1.10	1.14	1.09
3	4	6	4	7	4.42	4.41	4.56	4.16
4	64	64	66	74	2.51	2.80	2.50	2.23
5	75	66	70	68	1.96	2.07	2.06	2.11
6	15	13	12	12	4.47	4.45	4.63	4.61
7	99	99	99	99	0.63	0.66	0.79	0.83
8	98	98	97	99	0.76	1.00	0.89	1.03
9	100	100	99	100	0.58	0.67	0.63	0.69
10	51	49	48	48	2.59	2.87	2.75	2.76
11	94	93	94	97	1.12	1.31	1.16	1.07
12	96	96	99	96	0.85	0.84	0.79	0.87
13	43	30	36	32	2.61	2.83	2.85	2.80
14	64	68	59	68	2.43	2.18	2.39	2.12
15	95	89	92	92	1.18	1.38	1.33	1.36
16	19	24	26	22	6.51	6.04	5.76	6.16
17	88	89	88	86	1.43	1.49	1.51	1.48
18	63	59	69	66	2.25	2.38	2.04	2.07
19	86	81	81	81	1.57	1.73	1.58	1.55

SDR [%], successful detection rate within a given radius; MRE [mm], mean radial error. The study tackled 4 algorithms: ALD; CNeg, contrasted-negative augmented; Con, contrast augmented; Neg, negative augmented.

**Table 6:** Experimental results involved the detection of 83 cephalometric points across 215 test images, reporting the percentage of correctly localized points within a 2.5 mm threshold and the mean deviation from the ground truth (in mm).

pt	SDR 2.5 mm [%]				MRE [mm]			
	ALD	CNeg	Con	Neg	ALD	CNeg	Con	Neg
1	67.44	66.05	49.30	51.63	2.25	2.22	3.24	2.70
2	73.49	70.70	48.84	65.12	1.98	2.05	3.17	2.35
3	48.37	53.02	30.70	43.26	3.10	2.90	4.23	3.26
4	69.77	61.86	52.09	66.05	2.14	2.39	3.41	2.27
5	88.37	82.79	61.86	66.51	1.52	1.65	2.93	2.22
6	55.35	56.28	38.14	57.21	2.85	2.74	3.93	2.72
7	31.63	41.40	26.05	32.56	4.02	3.72	4.79	4.04
8	62.33	62.33	46.51	61.40	2.39	2.65	3.52	2.70
9	77.67	80.93	41.86	80.47	1.85	1.67	2.96	1.74
10	52.09	55.81	38.14	49.77	2.91	2.66	3.87	3.14
11	71.63	72.09	57.67	73.02	2.12	2.05	3.15	2.09
12	84.19	78.60	60.47	81.86	1.56	1.79	2.53	1.58
13	66.51	68.37	27.44	57.21	2.26	2.24	3.94	2.49
14	88.84	92.56	54.88	93.49	1.29	1.12	3.11	1.09
15	56.74	59.53	49.77	59.53	2.67	2.72	3.40	2.76
16	82.79	84.19	54.42	82.79	1.71	1.69	3.13	1.63
17	80.00	90.23	60.93	86.98	1.75	1.32	2.76	1.60
18	64.65	56.74	46.05	59.53	2.54	2.63	3.56	2.62
19	59.07	64.65	42.33	56.74	2.73	2.66	3.55	2.92
20	57.21	61.86	53.02	65.58	2.78	2.59	3.13	2.54
21	69.30	83.26	38.60	75.81	2.40	1.79	4.10	1.89
22	82.79	82.79	50.23	85.58	1.65	1.63	3.13	1.52
23	64.19	63.26	45.12	64.65	2.33	2.46	3.45	2.49
24	80.00	89.77	46.05	82.79	1.74	1.46	3.05	1.69
25	92.09	92.09	53.49	90.23	1.21	1.24	3.24	1.39
26	77.21	84.19	53.02	80.47	1.86	1.62	3.00	1.73
27	83.26	81.86	70.70	80.00	1.70	1.73	2.23	1.72
28	56.28	48.37	34.88	60.00	2.63	2.90	4.03	2.62
29	91.63	91.16	66.51	93.02	1.32	1.32	2.66	1.30
30	70.70	65.12	47.44	63.72	2.28	2.49	3.47	2.67
31	46.98	45.58	34.88	49.30	3.22	3.50	4.20	3.16
32	93.95	94.42	58.60	94.88	1.24	1.13	3.08	1.16
33	87.91	92.56	66.98	82.79	1.57	1.27	2.19	1.80
34	70.23	71.63	42.79	64.19	2.09	2.11	3.59	2.36
35	65.12	66.98	50.70	62.33	2.53	2.49	3.36	2.60
36	58.60	49.30	35.35	52.56	2.72	3.00	4.28	3.13
37	55.81	57.21	41.86	54.42	2.67	2.85	3.58	2.89
38	69.77	66.51	45.12	68.37	2.13	2.22	3.64	2.55
39	84.19	84.65	60.47	83.26	1.76	1.77	2.66	1.85
40	85.58	86.05	27.91	84.19	1.56	1.64	4.12	1.54
41	77.67	85.12	33.02	83.72	1.91	1.63	4.19	1.75
42	66.05	76.74	32.56	75.35	2.34	1.96	4.16	2.06
43	58.60	50.23	35.35	61.86	2.70	2.86	4.19	2.47
44	60.00	68.37	32.56	69.30	2.42	2.15	3.78	2.13
45	71.16	85.12	26.51	87.44	2.28	1.68	4.60	1.47
46	78.60	82.79	52.56	82.33	1.74	1.69	2.89	1.58
47	81.86	77.67	52.09	84.65	1.70	1.89	2.81	1.56
48	50.23	53.95	25.58	45.12	3.25	2.79	4.84	3.18
49	78.14	84.19	45.12	83.72	1.74	1.55	3.12	1.51
50	90.70	86.98	66.05	93.02	1.40	1.41	2.56	1.26
51	60.93	56.74	51.63	57.21	2.53	2.72	3.22	2.73
52	64.19	60.47	45.58	59.53	2.55	2.68	3.56	2.81
53	63.26	69.77	55.35	61.40	2.55	2.11	3.24	2.55
54	76.74	80.00	59.53	79.53	2.13	1.83	2.85	1.85
55	81.86	84.65	57.67	90.23	1.78	1.59	2.74	1.46
56	62.79	60.93	45.58	59.53	2.45	2.70	3.39	2.68
57	90.70	95.35	68.37	94.42	1.24	1.11	2.37	1.04
58	48.37	49.77	40.47	47.44	3.11	3.06	3.71	3.22
59	63.26	61.86	43.72	59.07	2.67	2.54	3.82	2.58
60	41.86	46.05	37.21	51.16	3.31	3.05	3.98	2.97
61	57.21	55.81	43.26	50.70	2.49	2.77	3.57	3.00
62	70.70	66.05	49.30	64.65	2.26	2.51	3.44	2.38
63	54.88	63.26	46.51	56.28	2.73	2.59	3.61	2.61
64	59.07	63.72	45.58	52.56	2.66	2.49	3.48	2.71
65	76.74	72.56	57.67	75.81	1.86	2.06	3.01	1.98
66	80.47	80.00	56.74	79.53	1.77	1.85	2.86	1.90
67	84.65	86.05	61.86	90.70	1.54	1.55	2.53	1.46
68	65.58	66.51	48.84	62.33	2.34	2.26	3.35	2.40
69	48.37	43.72	39.53	35.81	2.85	3.08	3.96	3.30
70	63.72	65.58	48.37	58.60	2.40	2.47	3.44	2.66
71	76.74	77.21	53.95	72.56	1.87	1.88	3.04	2.05
72	72.56	71.16	52.09	72.56	2.01	2.15	3.20	2.21
73	75.81	69.77	52.09	72.56	2.02	2.22	3.35	2.18
74	72.09	76.28	58.60	76.74	2.10	1.97	2.85	2.05
75	72.56	77.67	50.70	73.95	2.05	1.87	3.17	1.89
76	60.93	71.63	59.53	67.91	2.54	2.11	3.00	2.23
77	29.77	33.95	21.40	27.91	4.84	4.73	5.43	4.62
78	30.23	32.09	21.86	34.42	4.26	4.21	5.38	4.08
79	86.51	87.91	59.07	87.44	1.52	1.44	2.78	1.49
80	80.93	81.86	58.14	84.65	1.83	1.68	2.86	1.58
81	62.33	68.37	46.98	63.72	2.38	2.18	3.35	2.31
82	76.74	80.47	60.93	81.40	1.88	1.70	2.73	1.71
83	66.51	63.26	44.19	63.72	2.24	2.33	3.50	2.43

SDR [%], successful detection rate within a given radius; MRE [mm], mean radial error. The study tackles 4 algorithms: ALD; CNeg, contrasted-negative augmented; Con, contrast augmented; Neg, negative augmented.

## Data Availability

The data presented in this study are available on request from the corresponding author. The data are not publicly available due to the privacy reasons.

## Supplementary Materials

The following supporting information can be downloaded at: https://www.mdpi.com/article/10.3390/diagnostics16111638/s1, File S1. Code Implementation and Training Details for the Automated Cephalometric Points Marking System.
