# Supplementary material for: Automated Cephalometric Points Marking System
Source: Diagnostics (Basel). 2026 May 27;16(11):1638. doi: 10.3390/diagnostics16111638 (PMC13257313; doi:10.3390/diagnostics16111638)
Supplement: Supplementary file 1 [file diagnostics-16-01638-s001.zip › diagnostics-4048353-supplementary.pdf]

Supplementary Material: Code Implementation and Training Details for the Automated Cephalometric Points Marking System

Table of Contents

INTRODUCTION 2

REPOSITORY STRUCTURE 2

SYSTEM REQUIREMENTS 2

DATASET 3

IMAGE AUGMENTATION 3

TRAINING 3

PREDICTION 3

REPRODUCIBILITY 3

NOTES 4

Introduction

This supplementary document provides detailed information regarding the implementation, training configuration, and usage of the source code associated with the Automated Cephalometric Points Marking System. The codebase corresponding to this work is publicly available in the following GitHub repository:

https://github.com/erickosmala/supplemental_code_for_automated_cephalometric_points_marking_system

This document is intended to complement the main manuscript by providing additional technical details that facilitate reproducibility and implementation of the proposed approach.

Repository Structure

The repository is organized as follows:

- ALD/ – contains the implementation of the baseline solution on which the proposed tool is built.
- data/ – contains the dataset used in the experiments. This directory includes:
  - images/ – cephalometric X-ray images used as input data
  - annotations / landmark labels – ground-truth coordinates of cephalometric points
- model/ – contains model trained to predict 19 cephalometric landmarks.
- models.py – defines the architectures of the selected baseline models used in the study.
- train_model.py – script responsible for training the models.
- predict.py – script used for inference and evaluation.
- image_augmentation.py – contains all image augmentation techniques used to create additional training subsets.
- config.py – contains configuration settings for training and evaluation.
- requirements.txt – lists all dependencies required to reproduce the environment and run the code.

System Requirements

The implementation was developed using the following environment:

- Python 3.10

- All required libraries can be found in the requirements.txt file

Dataset

The data/images_to_process folder contains a collection of images derived from the public Image_Annotation_EvaluationCode collection. In addition, there are 4 400_senior_*.csv files in the data folder, which contain the prepared information needed to train the model. Each file contains information about the location of a landmarks. In addition, in the config.py file it is possible to change selected parameters.

Image Augmentation

In order to create the image collections described in the mentioned article, use the image_augumentation.py script, in which we indicate the locations of the folder containing the images to be augmented next.

Training

To perform the process of training the model on the proposed dataset, run the train_model.py script. Information about the data for the training process itself can be found in the config.py file.

Prediction

In order to detect the location of landmarks for a given set of images, you need to run the predict.py script. In this script you need to specify the path to the model, by default it is set to the base model discussed in the article. Information about the data for the prediction process can be found in the config.py file.

Reproducibility

To ensure reproducibility:

1. Clone the repository from GitHub
2. Install all required dependencies
3. Prepare the dataset according to the described structure
4. Configure the experiment parameters

5. Run the training and evaluation scripts

All experiments described in the main manuscript can be reproduced using the provided codebase and configurations.

Notes

The GitHub repository contains the complete implementation of the proposed system.

This supplementary document provides a static reference to complement the dynamic codebase.

In case of updates to the repository, the latest version should be considered the authoritative implementation.
